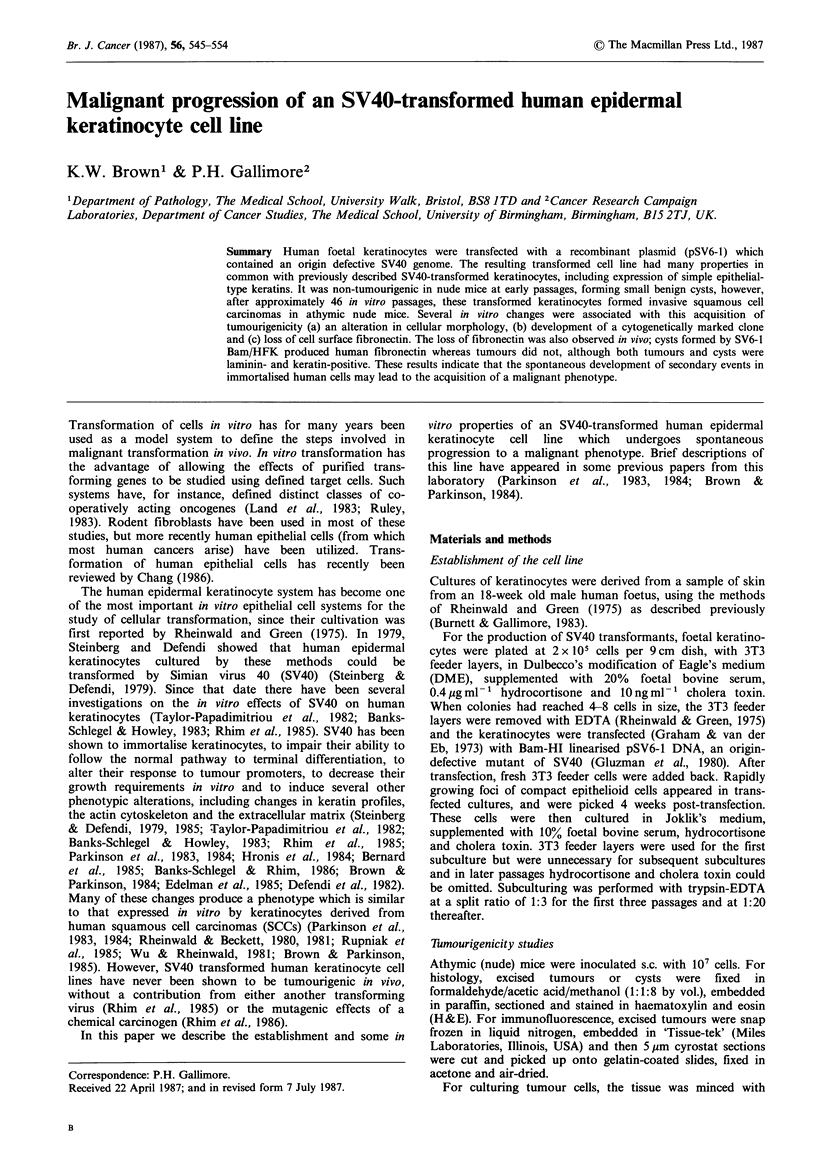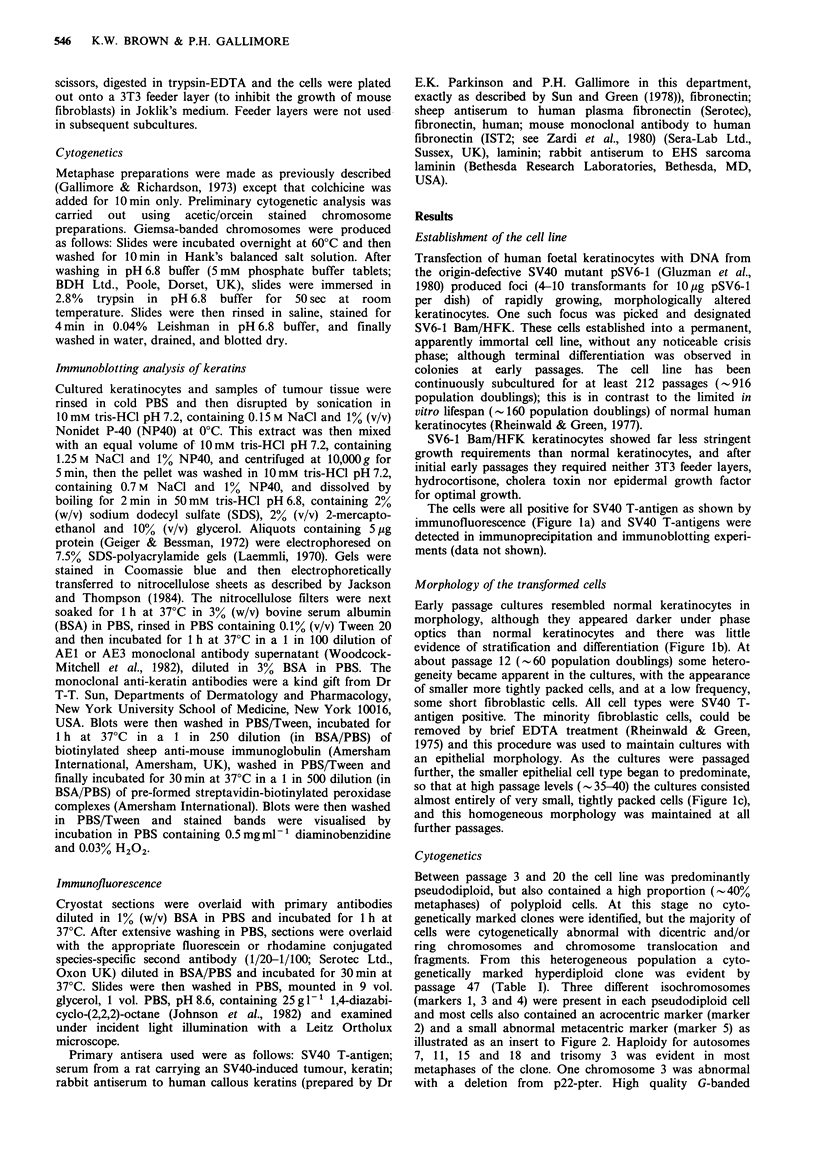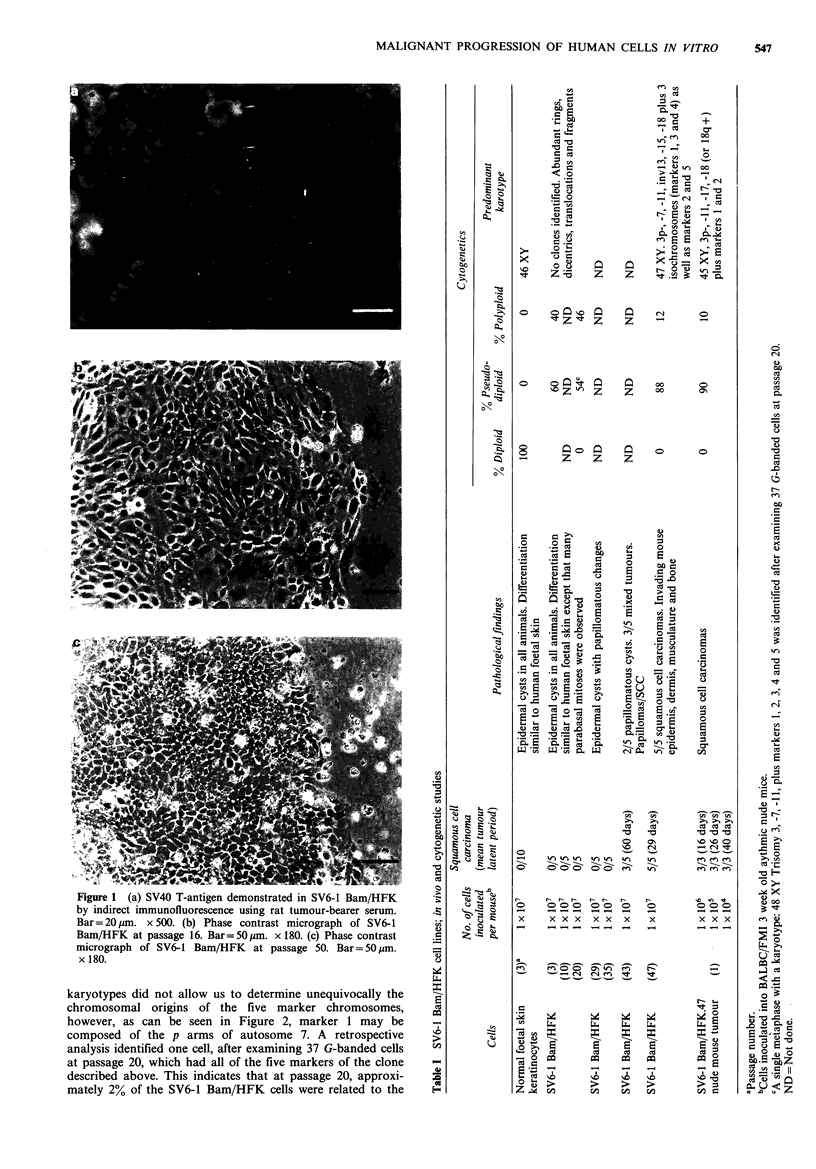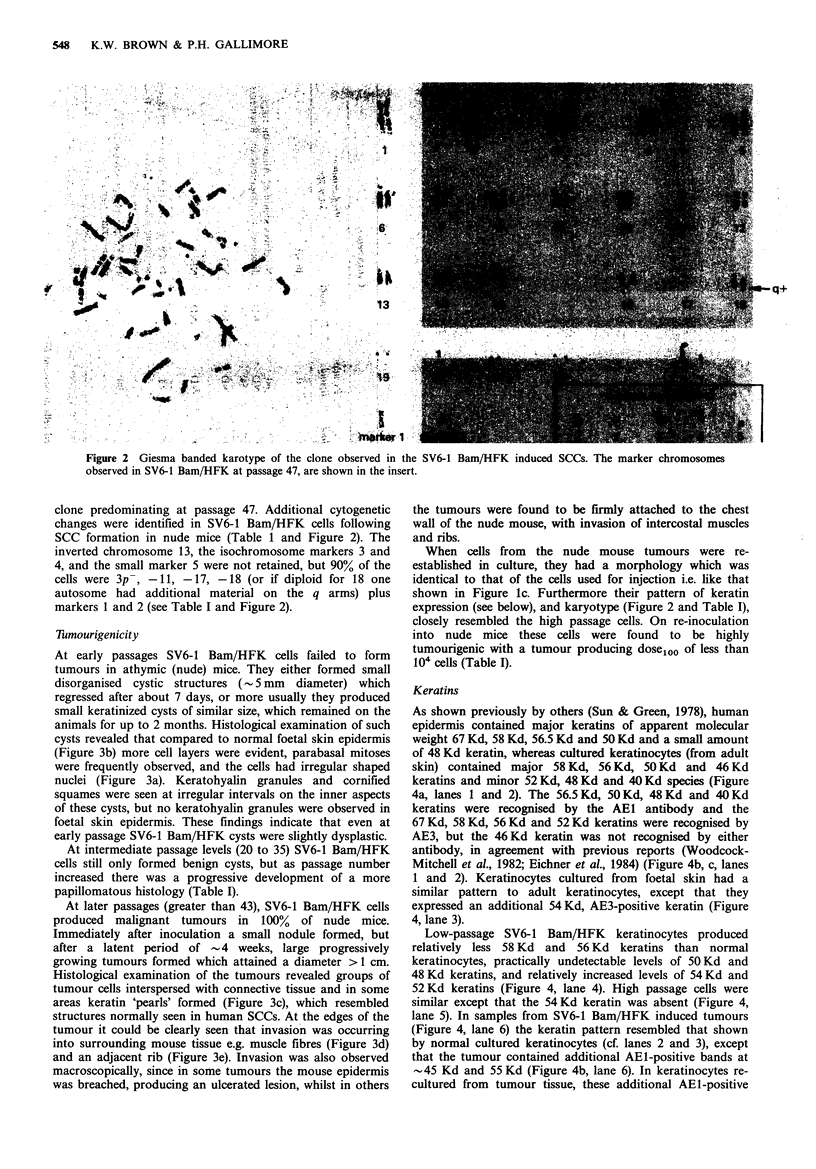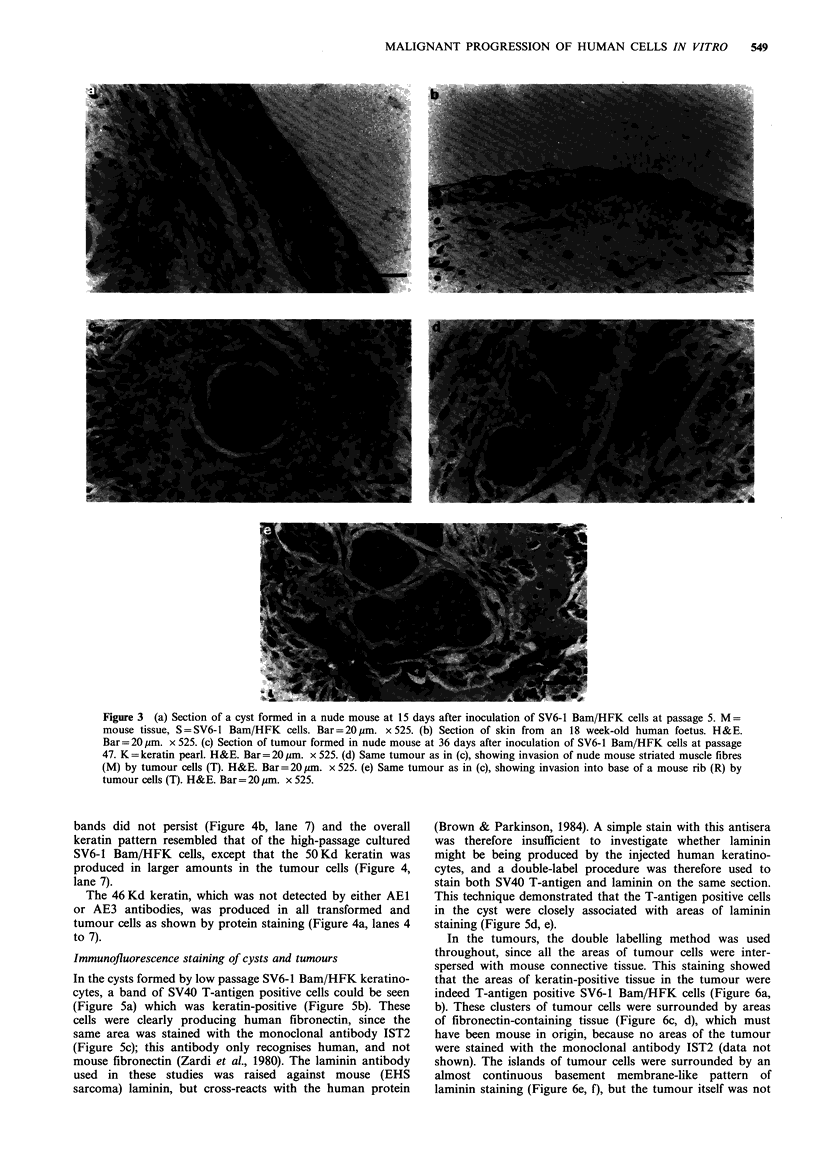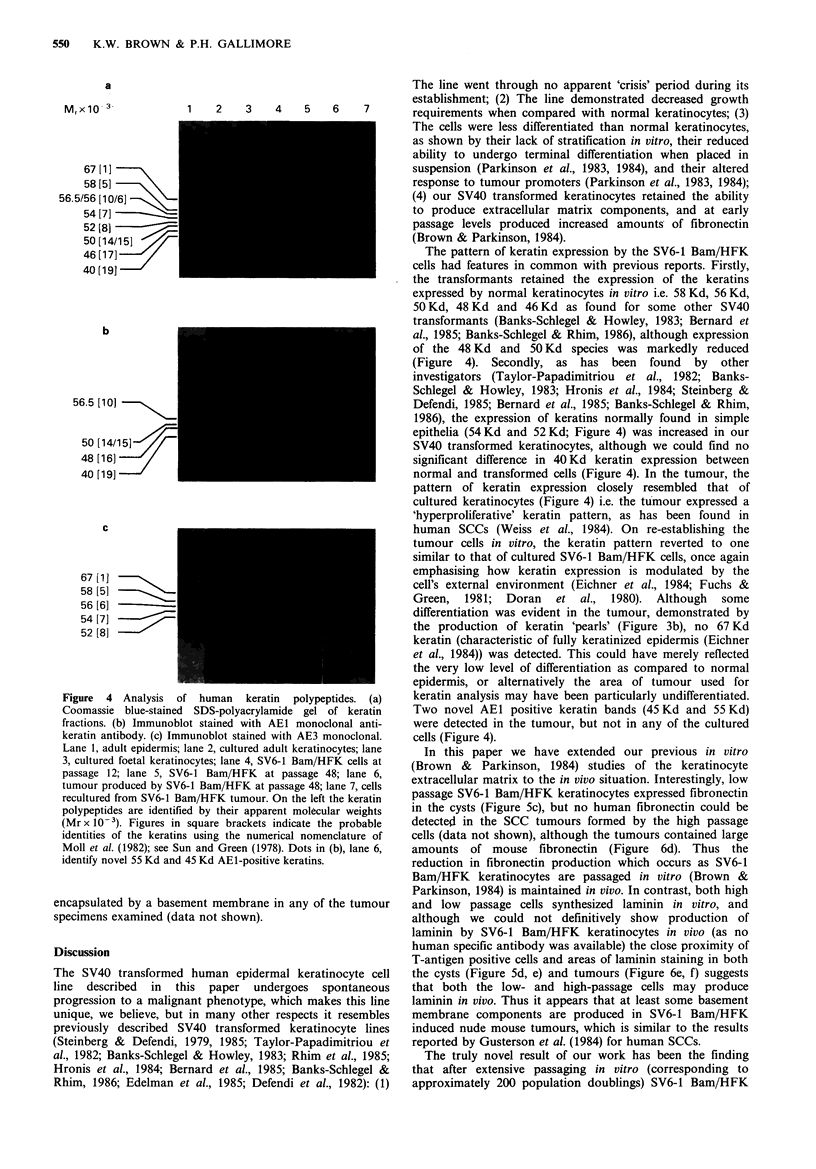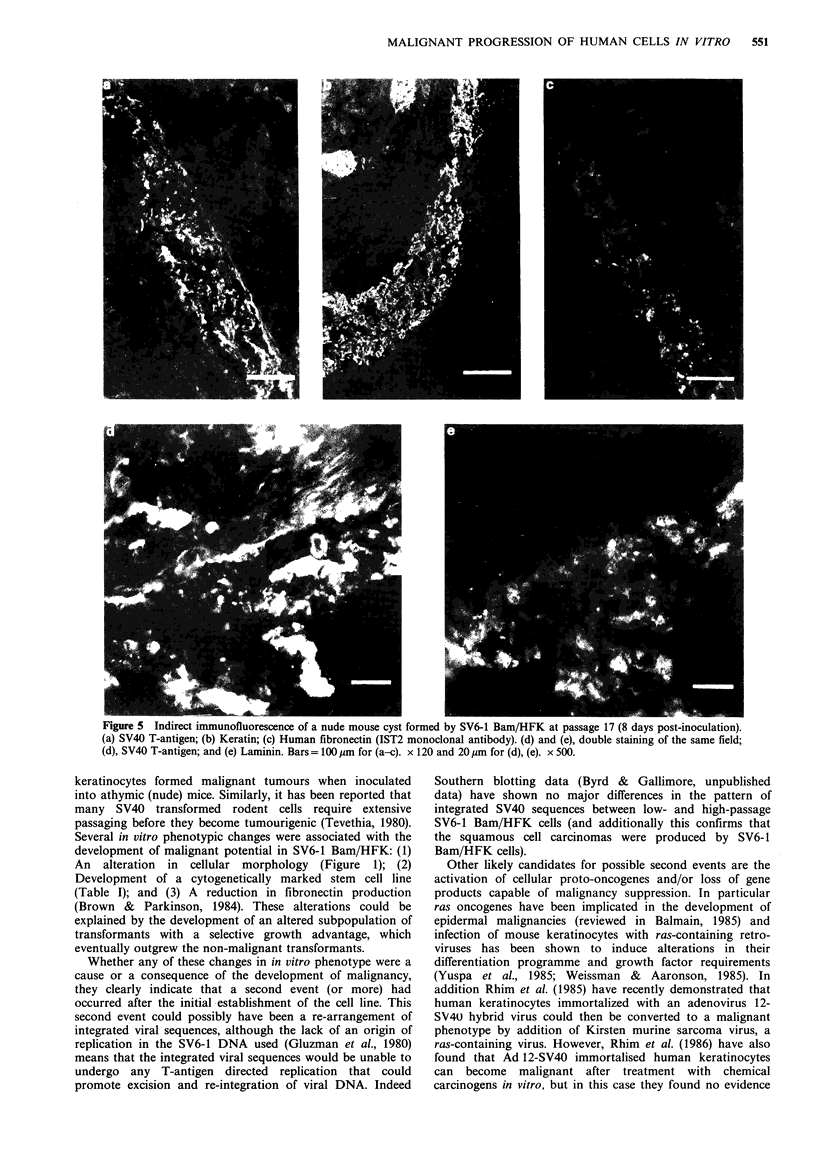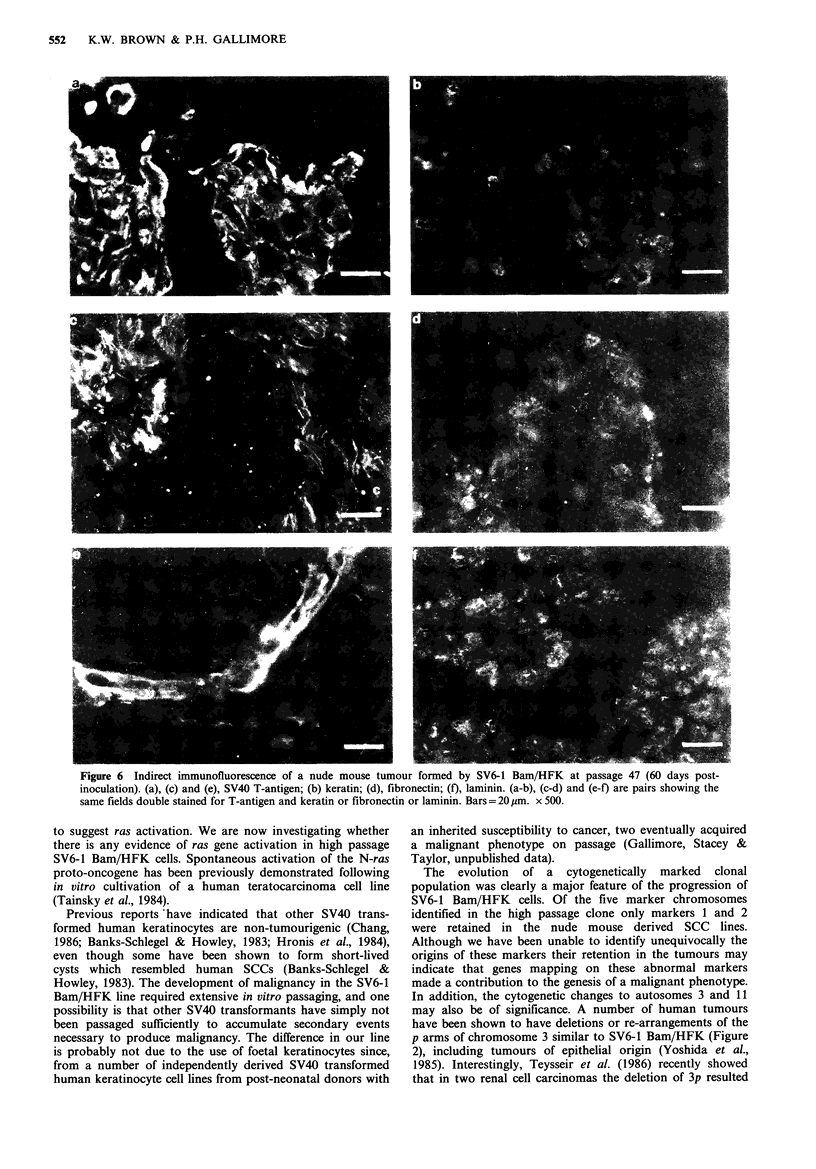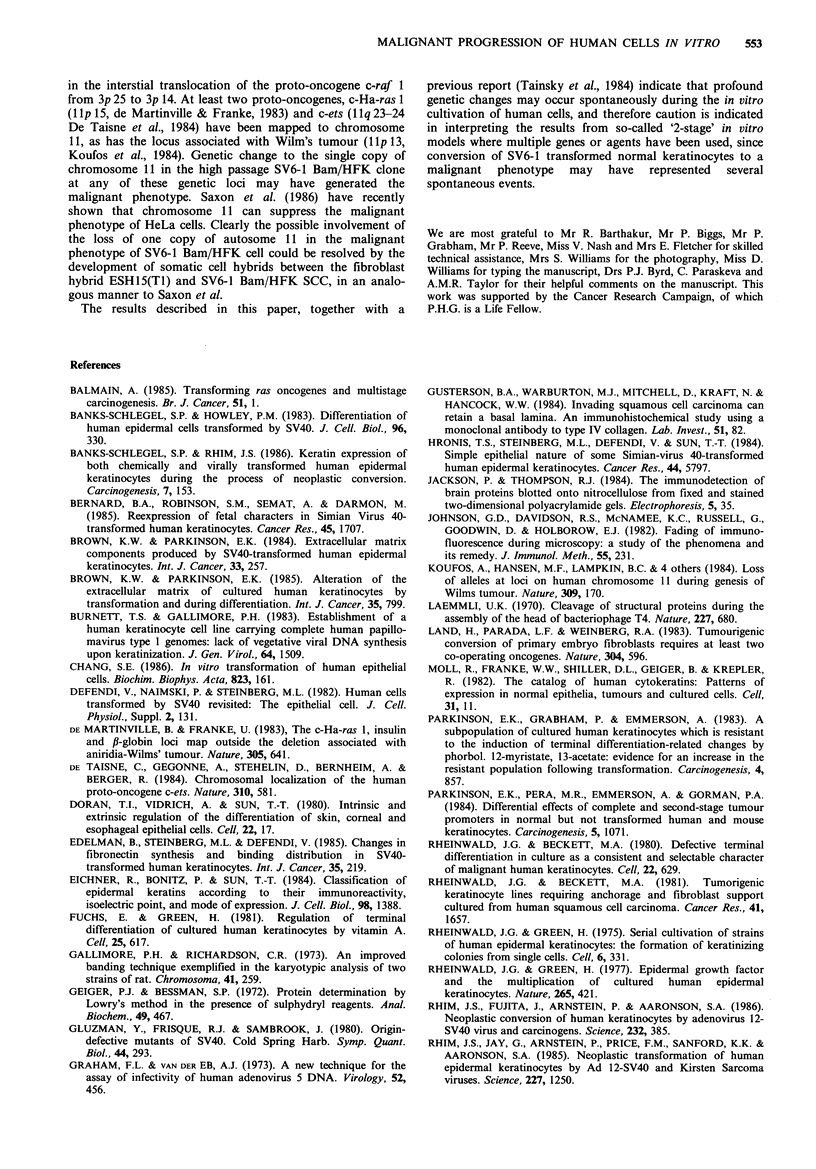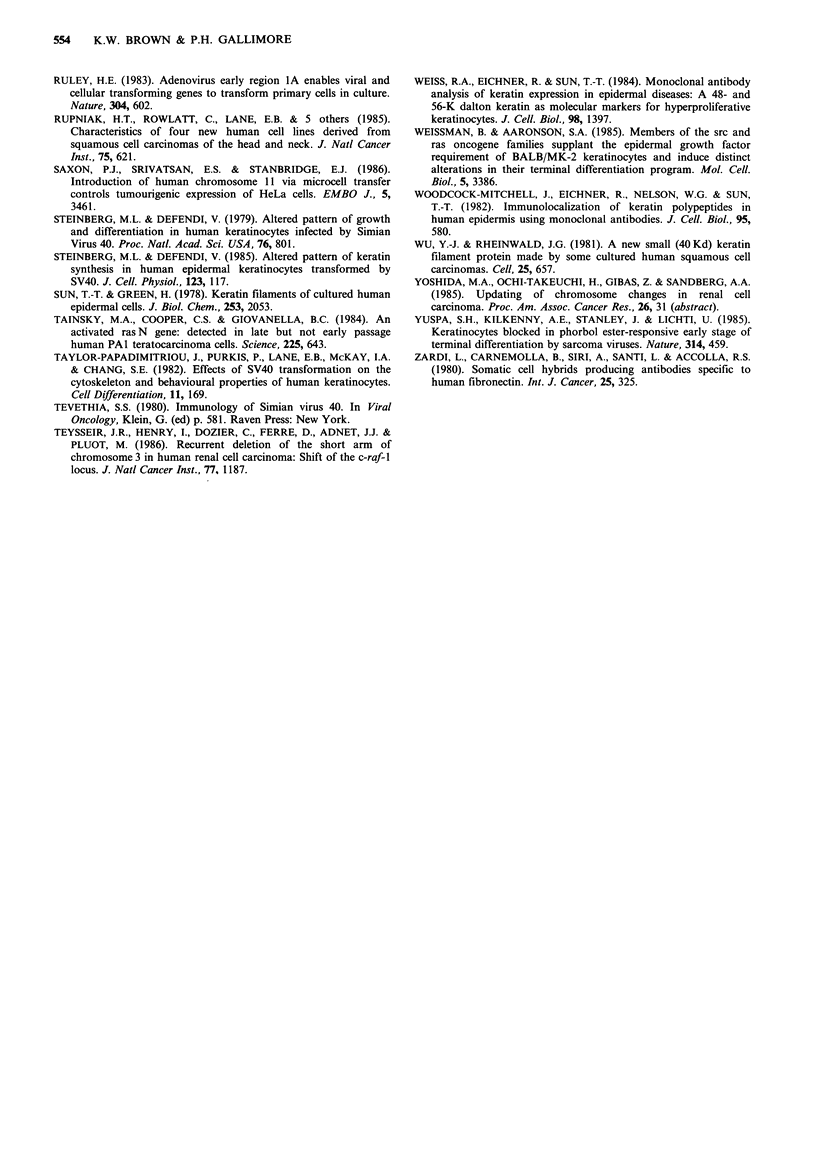# Malignant progression of an SV40-transformed human epidermal keratinocyte cell line.

**DOI:** 10.1038/bjc.1987.240

**Published:** 1987-11

**Authors:** K. W. Brown, P. H. Gallimore

**Affiliations:** Department of Pathology, Medical School, Bristol, UK.

## Abstract

**Images:**


*Br. J. Cancer* (1987), **56**, 545–554 © The Macmillan Press Ltd., 1987

### Malignant progression of an SV40-transformed human epidermal keratinocyte cell line

K.W. Brown[1] & P.H. Gallimore[2]

[1]*Department of Pathology, The Medical School, University Walk, Bristol, BS8 1TD and* [2]*Cancer Research Campaign Laboratories, Department of Cancer Studies, The Medical School, University of Birmingham, Birmingham, B15 2TJ, UK.*

**Summary** Human foetal keratinocytes were transfected with a recombinant plasmid (pSV6-1) which contained an origin defective SV40 genome. The resulting transformed cell line had many properties in common with previously described SV40-transformed keratinocytes, including expression of simple epithelial-type keratins. It was non-tumourigenic in nude mice at early passages, forming small benign cysts, however, after approximately 46 *in vitro* passages, these transformed keratinocytes formed invasive squamous cell carcinomas in athymic nude mice. Several *in vitro* changes were associated with this acquisition of tumourigenicity (a) an alteration in cellular morphology, (b) development of a cytogenetically marked clone and (c) loss of cell surface fibronectin. The loss of fibronectin was also observed *in vivo*; cysts formed by SV6-1 Bam/HFK produced human fibronectin whereas tumours did not, although both tumours and cysts were laminin- and keratin-positive. These results indicate that the spontaneous development of secondary events in immortalised human cells may lead to the acquisition of a malignant phenotype.

Transformation of cells *in vitro* has for many years been used as a model system to define the steps involved in malignant transformation *in vivo*. *In vitro* transformation has the advantage of allowing the effects of purified transforming genes to be studied using defined target cells. Such systems have, for instance, defined distinct classes of co-operatively acting oncogenes (Land *et al.*, 1983; Ruley, 1983). Rodent fibroblasts have been used in most of these studies, but more recently human epithelial cells (from which most human cancers arise) have been utilized. Transformation of human epithelial cells has recently been reviewed by Chang (1986).

The human epidermal keratinocyte system has become one of the most important *in vitro* epithelial cell systems for the study of cellular transformation, since their cultivation was first reported by Rheinwald and Green (1975). In 1979, Steinberg and Defendi showed that human epidermal keratinocytes cultured by these methods could be transformed by Simian virus 40 (SV40) (Steinberg & Defendi, 1979). Since that date there have been several investigations on the *in vitro* effects of SV40 on human keratinocytes (Taylor-Papadimitriou *et al.*, 1982; Banks-Schlegel & Howley, 1983; Rhim *et al.*, 1985). SV40 has been shown to immortalise keratinocytes, to impair their ability to follow the normal pathway to terminal differentiation, to alter their response to tumour promoters, to decrease their growth requirements *in vitro* and to induce several other phenotypic alterations, including changes in keratin profiles, the actin cytoskeleton and the extracellular matrix (Steinberg & Defendi, 1979, 1985; Taylor-Papadimitriou *et al.*, 1982; Banks-Schlegel & Howley, 1983; Rhim *et al.*, 1985; Parkinson *et al.*, 1983, 1984; Hronis *et al.*, 1984; Bernard *et al.*, 1985; Banks-Schlegel & Rhim, 1986; Brown & Parkinson, 1984; Edelman *et al.*, 1985; Defendi *et al.*, 1982). Many of these changes produce a phenotype which is similar to that expressed *in vitro* by keratinocytes derived from human squamous cell carcinomas (SCCs) (Parkinson *et al.*, 1983, 1984; Rheinwald & Beckett, 1980, 1981; Rupniak *et al.*, 1985; Wu & Rheinwald, 1981; Brown & Parkinson, 1985). However, SV40 transformed human keratinocyte cell lines have never been shown to be tumourigenic *in vivo*, without a contribution from either another transforming virus (Rhim *et al.*, 1985) or the mutagenic effects of a chemical carcinogen (Rhim *et al.*, 1986).

In this paper we describe the establishment and some *in vitro* properties of an SV40-transformed human epidermal keratinocyte cell line which undergoes spontaneous progression to a malignant phenotype. Brief descriptions of this line have appeared in some previous papers from this laboratory (Parkinson *et al.*, 1983, 1984; Brown & Parkinson, 1984).

Correspondence: P.H. Gallimore.
Received 22 April 1987; and in revised form 7 July 1987.

### Materials and methods

### Establishment of the cell line

Cultures of keratinocytes were derived from a sample of skin from an 18-week old male human foetus, using the methods of Rheinwald and Green (1975) as described previously (Burnett & Gallimore, 1983).

For the production of SV40 transformants, foetal keratinocytes were plated at $2\times10^5$ cells per 9 cm dish, with 3T3 feeder layers, in Dulbecco's modification of Eagle's medium (DME), supplemented with 20% foetal bovine serum, $0.4\,\mu g\,ml^{-1}$ hydrocortisone and $10\,ng\,ml^{-1}$ cholera toxin. When colonies had reached 4–8 cells in size, the 3T3 feeder layers were removed with EDTA (Rheinwald & Green, 1975) and the keratinocytes were transfected (Graham & van der Eb, 1973) with Bam-HI linearised pSV6-1 DNA, an origin-defective mutant of SV40 (Gluzman *et al.*, 1980). After transfection, fresh 3T3 feeder cells were added back. Rapidly growing foci of compact epithelioid cells appeared in transfected cultures, and were picked 4 weeks post-transfection. These cells were then cultured in Joklik's medium, supplemented with 10% foetal bovine serum, hydrocortisone and cholera toxin. 3T3 feeder layers were used for the first subculture but were unnecessary for subsequent subcultures and in later passages hydrocortisone and cholera toxin could be omitted. Subculturing was performed with trypsin-EDTA at a split ratio of 1:3 for the first three passages and at 1:20 thereafter.

### Tumourigenicity studies

Athymic (nude) mice were inoculated s.c. with $10^7$ cells. For histology, excised tumours or cysts were fixed in formaldehyde/acetic acid/methanol (1:1:8 by vol.), embedded in paraffin, sectioned and stained in haematoxylin and eosin (H&E). For immunofluorescence, excised tumours were snap frozen in liquid nitrogen, embedded in 'Tissue-tek' (Miles Laboratories, Illinois, USA) and then $5\,\mu m$ cyrostat sections were cut and picked up onto gelatin-coated slides, fixed in acetone and air-dried.

For culturing tumour cells, the tissue was minced with scissors, digested in trypsin-EDTA and the cells were plated out onto a 3T3 feeder layer (to inhibit the growth of mouse fibroblasts) in Joklik's medium. Feeder layers were not used in subsequent subcultures.

*Cytogenetics*

Metaphase preparations were made as previously described (Gallimore & Richardson, 1973) except that colchicine was added for 10 min only. Preliminary cytogenetic analysis was carried out using acetic/orcein stained chromosome preparations. Giemsa-banded chromosomes were produced as follows: Slides were incubated overnight at 60°C and then washed for 10 min in Hank's balanced salt solution. After washing in pH 6.8 buffer (5 mM phosphate buffer tablets; BDH Ltd., Poole, Dorset, UK), slides were immersed in 2.8% trypsin in pH 6.8 buffer for 50 sec at room temperature. Slides were then rinsed in saline, stained for 4 min in 0.04% Leishman in pH 6.8 buffer, and finally washed in water, drained, and blotted dry.

*Immunoblotting analysis of keratins*

Cultured keratinocytes and samples of tumour tissue were rinsed in cold PBS and then disrupted by sonication in 10 mM tris-HCl pH 7.2, containing 0.15 M NaCl and 1% (v/v) Nonidet P-40 (NP40) at 0°C. This extract was then mixed with an equal volume of 10 mM tris-HCl pH 7.2, containing 1.25 M NaCl and 1% NP40, and centrifuged at 10,000 *g* for 5 min, then the pellet was washed in 10 mM tris-HCl pH 7.2, containing 0.7 M NaCl and 1% NP40, and dissolved by boiling for 2 min in 50 mM tris-HCl pH 6.8, containing 2% (w/v) sodium dodecyl sulfate (SDS), 2% (v/v) 2-mercapto-ethanol and 10% (v/v) glycerol. Aliquots containing 5 $\mu$g protein (Geiger & Bessman, 1972) were electrophoresed on 7.5% SDS-polyacrylamide gels (Laemmli, 1970). Gels were stained in Coomassie blue and then electrophoretically transferred to nitrocellulose sheets as described by Jackson and Thompson (1984). The nitrocellulose filters were next soaked for 1 h at 37°C in 3% (w/v) bovine serum albumin (BSA) in PBS, rinsed in PBS containing 0.1% (v/v) Tween 20 and then incubated for 1 h at 37°C in a 1 in 100 dilution of AE1 or AE3 monoclonal antibody supernatant (Woodcock-Mitchell *et al.*, 1982), diluted in 3% BSA in PBS. The monoclonal anti-keratin antibodies were a kind gift from Dr T-T. Sun, Departments of Dermatology and Pharmacology, New York University School of Medicine, New York 10016, USA. Blots were then washed in PBS/Tween, incubated for 1 h at 37°C in a 1 in 250 dilution (in BSA/PBS) of biotinylated sheep anti-mouse immunoglobulin (Amersham International, Amersham, UK), washed in PBS/Tween and finally incubated for 30 min at 37°C in a 1 in 500 dilution (in BSA/PBS) of pre-formed streptavidin-biotinylated peroxidase complexes (Amersham International). Blots were then washed in PBS/Tween and stained bands were visualised by incubation in PBS containing 0.5 mg ml$^{-1}$ diaminobenzidine and 0.03% $H_2O_2$.

*Immunofluorescence*

Cryostat sections were overlaid with primary antibodies diluted in 1% (w/v) BSA in PBS and incubated for 1 h at 37°C. After extensive washing in PBS, sections were overlaid with the appropriate fluorescein or rhodamine conjugated species-specific second antibody (1/20–1/100; Serotec Ltd., Oxon UK) diluted in BSA/PBS and incubated for 30 min at 37°C. Slides were then washed in PBS, mounted in 9 vol. glycerol, 1 vol. PBS, pH 8.6, containing 25 g l$^{-1}$ 1,4-diazabi-cyclo-(2,2,2)-octane (Johnson *et al.*, 1982) and examined under incident light illumination with a Leitz Ortholux microscope.

Primary antisera used were as follows: SV40 T-antigen; serum from a rat carrying an SV40-induced tumour, keratin; rabbit antiserum to human callous keratins (prepared by Dr E.K. Parkinson and P.H. Gallimore in this department, exactly as described by Sun and Green (1978)), fibronectin; sheep antiserum to human plasma fibronectin (Serotec), fibronectin, human; mouse monoclonal antibody to human fibronectin (IST2; see Zardi *et al.*, 1980) (Sera-Lab Ltd., Sussex, UK), laminin; rabbit antiserum to EHS sarcoma laminin (Bethesda Research Laboratories, Bethesda, MD, USA).

### Results

*Establishment of the cell line*

Transfection of human foetal keratinocytes with DNA from the origin-defective SV40 mutant pSV6-1 (Gluzman *et al.*, 1980) produced foci (4–10 transformants for 10 $\mu$g pSV6-1 per dish) of rapidly growing, morphologically altered keratinocytes. One such focus was picked and designated SV6-1 Bam/HFK. These cells established into a permanent, apparently immortal cell line, without any noticeable crisis phase; although terminal differentiation was observed in colonies at early passages. The cell line has been continuously subcultured for at least 212 passages ($\sim$916 population doublings); this is in contrast to the limited *in vitro* lifespan ($\sim$160 population doublings) of normal human keratinocytes (Rheinwald & Green, 1977).

SV6-1 Bam/HFK keratinocytes showed far less stringent growth requirements than normal keratinocytes, and after initial early passages they required neither 3T3 feeder layers, hydrocortisone, cholera toxin nor epidermal growth factor for optimal growth.

The cells were all positive for SV40 T-antigen as shown by immunofluorescence (Figure 1a) and SV40 T-antigens were detected in immunoprecipitation and immunoblotting experiments (data not shown).

*Morphology of the transformed cells*

Early passage cultures resembled normal keratinocytes in morphology, although they appeared darker under phase optics than normal keratinocytes and there was little evidence of stratification and differentiation (Figure 1b). At about passage 12 ($\sim$60 population doublings) some heterogeneity became apparent in the cultures, with the appearance of smaller more tightly packed cells, and at a low frequency, some short fibroblastic cells. All cell types were SV40 T-antigen positive. The minority fibroblastic cells, could be removed by brief EDTA treatment (Rheinwald & Green, 1975) and this procedure was used to maintain cultures with an epithelial morphology. As the cultures were passaged further, the smaller epithelial cell type began to predominate, so that at high passage levels ($\sim$35–40) the cultures consisted almost entirely of very small, tightly packed cells (Figure 1c), and this homogeneous morphology was maintained at all further passages.

*Cytogenetics*

Between passage 3 and 20 the cell line was predominantly pseudodiploid, but also contained a high proportion ($\sim$40% metaphases) of polyploid cells. At this stage no cytogenetically marked clones were identified, but the majority of cells were cytogenetically abnormal with dicentric and/or ring chromosomes and chromosome translocation and fragments. From this heterogeneous population a cytogenetically marked hyperdiploid clone was evident by passage 47 (Table I). Three different isochromosomes (markers 1, 3 and 4) were present in each pseudodiploid cell and most cells also contained an acrocentric marker (marker 2) and a small abnormal metacentric marker (marker 5) as illustrated as an insert to Figure 2. Haploidy for autosomes 7, 11, 15 and 18 and trisomy 3 was evident in most metaphases of the clone. One chromosome 3 was abnormal with a deletion from p22-pter. High quality *G*-banded

**Figure 1** (a) SV40 T-antigen demonstrated in SV6-1 Bam/HFK by indirect immunofluorescence using rat tumour-bearer serum. Bar = 20 μm. ×500. (b) Phase contrast micrograph of SV6-1 Bam/HFK at passage 16. Bar = 50 μm. ×180. (c) Phase contrast micrograph of SV6-1 Bam/HFK at passage 50. Bar = 50 μm. ×180.

karyotypes did not allow us to determine unequivocally the chromosomal origins of the five marker chromosomes, however, as can be seen in Figure 2, marker 1 may be composed of the *p* arms of autosome 7. A retrospective analysis identified one cell, after examining 37 *G*-banded cells at passage 20, which had all of the five markers of the clone described above. This indicates that at passage 20, approximately 2% of the SV6-1 Bam/HFK cells were related to the

**Table I** SV6-1 Bam/HFK cell lines; *in vivo* and cytogenetic studies

| *Cells* | | *No. of cells inoculated per mouse*[b] | *Squamous cell carcinoma (mean tumour latent period)* | *Pathological findings* | *Cytogenetics* | | | |
|---|---|---|---|---|---|---|---|---|
| | | | | | *% Diploid* | *% Pseudo-diploid* | *% Polyploid* | *Predominant karotype* |
| Normal foetal skin keratinocytes | (3)[a] | $1\times10^7$ | 0/10 | Epidermal cysts in all animals. Differentiation similar to human foetal skin | 100 | 0 | 0 | 46 XY |
| SV6-1 Bam/HFK | (3) | $1\times10^7$ | 0/5 | Epidermal cysts in all animals. Differentiation similar to human foetal skin except that many parabasal mitoses were observed | | 60 | 40 | No clones identified. Abundant rings, dicentrics, translocations and fragments |
| | (10) | $1\times10^7$ | 0/5 | | ND | ND | ND | |
| | (20) | $1\times10^7$ | 0/5 | | 0 | 54[c] | 46 | |
| SV6-1 Bam/HFK | (29) | $1\times10^7$ | 0/5 | Epidermal cysts with papillomatous changes | ND | ND | ND | ND |
| | (35) | $1\times10^7$ | 0/5 | | | | | |
| SV6-1 Bam/HFK | (43) | $1\times10^7$ | 3/5 (60 days) | 2/5 papillomatous cysts. 3/5 mixed tumours. Papillomas/SCC | ND | ND | ND | ND |
| SV6-1 Bam/HFK | (47) | $1\times10^7$ | 5/5 (29 days) | 5/5 squamous cell carcinomas. Invading mouse epidermis, dermis, musculature and bone | 0 | 88 | 12 | 47 XY. 3p-, -7, -11, inv13, -15, -18 plus 3 isochromosomes (markers 1, 3 and 4) as well as markers 2 and 5 |
| SV6-1 Bam/HFK.47 nude mouse tumour | (1) | $1\times10^6$ | 3/3 (16 days) | Squamous cell carcinomas | 0 | 90 | 10 | 45 XY, 3p-, -11, -17, -18 (or 18q+) plus markers 1 and 2 |
| | | $1\times10^5$ | 3/3 (26 days) | | | | | |
| | | $1\times10^4$ | 3/3 (40 days) | | | | | |

[a]Passage number.

[b]Cells inoculated into BALBC/FMI 3 week old aythmic nude mice.

[c]A single metaphase with a karyotype: 48 XY Trisomy 3, -7, -11, plus markers 1, 2, 3, 4 and 5 was identified after examining 37 *G*-banded cells at passage 20.

ND = Not done.

**Figure 2** Giesma banded karotype of the clone observed in the SV6-1 Bam/HFK induced SCCs. The marker chromosomes observed in SV6-1 Bam/HFK at passage 47, are shown in the insert.

clone predominating at passage 47. Additional cytogenetic changes were identified in SV6-1 Bam/HFK cells following SCC formation in nude mice (Table 1 and Figure 2). The inverted chromosome 13, the isochromosome markers 3 and 4, and the small marker 5 were not retained, but 90% of the cells were $3p^-$, $-11$, $-17$, $-18$ (or if diploid for 18 one autosome had additional material on the *q* arms) plus markers 1 and 2 (see Table I and Figure 2).

*Tumourigenicity*

At early passages SV6-1 Bam/HFK cells failed to form tumours in athymic (nude) mice. They either formed small disorganised cystic structures ($\sim$5 mm diameter) which regressed after about 7 days, or more usually they produced small keratinized cysts of similar size, which remained on the animals for up to 2 months. Histological examination of such cysts revealed that compared to normal foetal skin epidermis (Figure 3b) more cell layers were evident, parabasal mitoses were frequently observed, and the cells had irregular shaped nuclei (Figure 3a). Keratohyalin granules and cornified squames were seen at irregular intervals on the inner aspects of these cysts, but no keratohyalin granules were observed in foetal skin epidermis. These findings indicate that even at early passage SV6-1 Bam/HFK cysts were slightly dysplastic.

At intermediate passage levels (20 to 35) SV6-1 Bam/HFK cells still only formed benign cysts, but as passage number increased there was a progressive development of a more papillomatous histology (Table I).

At later passages (greater than 43), SV6-1 Bam/HFK cells produced malignant tumours in 100% of nude mice. Immediately after inoculation a small nodule formed, but after a latent period of $\sim$4 weeks, large progressively growing tumours formed which attained a diameter $>1$ cm. Histological examination of the tumours revealed groups of tumour cells interspersed with connective tissue and in some areas keratin 'pearls' formed (Figure 3c), which resembled structures normally seen in human SCCs. At the edges of the tumour it could be clearly seen that invasion was occurring into surrounding mouse tissue e.g. muscle fibres (Figure 3d) and an adjacent rib (Figure 3e). Invasion was also observed macroscopically, since in some tumours the mouse epidermis was breached, producing an ulcerated lesion, whilst in others the tumours were found to be firmly attached to the chest wall of the nude mouse, with invasion of intercostal muscles and ribs.

When cells from the nude mouse tumours were re-established in culture, they had a morphology which was identical to that of the cells used for injection i.e. like that shown in Figure 1c. Furthermore their pattern of keratin expression (see below), and karyotype (Figure 2 and Table I), closely resembled the high passage cells. On re-inoculation into nude mice these cells were found to be highly tumourigenic with a tumour producing $dose_{100}$ of less than $10^4$ cells (Table I).

*Keratins*

As shown previously by others (Sun & Green, 1978), human epidermis contained major keratins of apparent molecular weight 67 Kd, 58 Kd, 56.5 Kd and 50 Kd and a small amount of 48 Kd keratin, whereas cultured keratinocytes (from adult skin) contained major 58 Kd, 56 Kd, 50 Kd and 46 Kd keratins and minor 52 Kd, 48 Kd and 40 Kd species (Figure 4a, lanes 1 and 2). The 56.5 Kd, 50 Kd, 48 Kd and 40 Kd keratins were recognised by the AE1 antibody and the 67 Kd, 58 Kd, 56 Kd and 52 Kd keratins were recognised by AE3, but the 46 Kd keratin was not recognised by either antibody, in agreement with previous reports (Woodcock-Mitchell *et al.*, 1982; Eichner *et al.*, 1984) (Figure 4b, c, lanes 1 and 2). Keratinocytes cultured from foetal skin had a similar pattern to adult keratinocytes, except that they expressed an additional 54 Kd, AE3-positive keratin (Figure 4, lane 3).

Low-passage SV6-1 Bam/HFK keratinocytes produced relatively less 58 Kd and 56 Kd keratins than normal keratinocytes, practically undetectable levels of 50 Kd and 48 Kd keratins, and relatively increased levels of 54 Kd and 52 Kd keratins (Figure 4, lane 4). High passage cells were similar except that the 54 Kd keratin was absent (Figure 4, lane 5). In samples from SV6-1 Bam/HFK induced tumours (Figure 4, lane 6) the keratin pattern resembled that shown by normal cultured keratinocytes (cf. lanes 2 and 3), except that the tumour contained additional AE1-positive bands at $\sim$45 Kd and 55 Kd (Figure 4b, lane 6). In keratinocytes re-cultured from tumour tissue, these additional AE1-positive

**Figure 3** (a) Section of a cyst formed in a nude mouse at 15 days after inoculation of SV6-1 Bam/HFK cells at passage 5. M = mouse tissue, S = SV6-1 Bam/HFK cells. Bar = 20 μm. × 525. (b) Section of skin from an 18 week-old human foetus. H&E. Bar = 20 μm. × 525. (c) Section of tumour formed in nude mouse at 36 days after inoculation of SV6-1 Bam/HFK cells at passage 47. K = keratin pearl. H&E. Bar = 20 μm. × 525. (d) Same tumour as in (c), showing invasion of nude mouse striated muscle fibres (M) by tumour cells (T). H&E. Bar = 20 μm. × 525. (e) Same tumour as in (c), showing invasion into base of a mouse rib (R) by tumour cells (T). H&E. Bar = 20 μm. × 525.

bands did not persist (Figure 4b, lane 7) and the overall keratin pattern resembled that of the high-passage cultured SV6-1 Bam/HFK cells, except that the 50 Kd keratin was produced in larger amounts in the tumour cells (Figure 4, lane 7).

The 46 Kd keratin, which was not detected by either AE1 or AE3 antibodies, was produced in all transformed and tumour cells as shown by protein staining (Figure 4a, lanes 4 to 7).

*Immunofluorescence staining of cysts and tumours*

In the cysts formed by low passage SV6-1 Bam/HFK keratinocytes, a band of SV40 T-antigen positive cells could be seen (Figure 5a) which was keratin-positive (Figure 5b). These cells were clearly producing human fibronectin, since the same area was stained with the monoclonal antibody IST2 (Figure 5c); this antibody only recognises human, and not mouse fibronectin (Zardi *et al.*, 1980). The laminin antibody used in these studies was raised against mouse (EHS sarcoma) laminin, but cross-reacts with the human protein (Brown & Parkinson, 1984). A simple stain with this antisera was therefore insufficient to investigate whether laminin might be being produced by the injected human keratinocytes, and a double-label procedure was therefore used to stain both SV40 T-antigen and laminin on the same section. This technique demonstrated that the T-antigen positive cells in the cyst were closely associated with areas of laminin staining (Figure 5d, e).

In the tumours, the double labelling method was used throughout, since all the areas of tumour cells were interspersed with mouse connective tissue. This staining showed that the areas of keratin-positive tissue in the tumour were indeed T-antigen positive SV6-1 Bam/HFK cells (Figure 6a, b). These clusters of tumour cells were surrounded by areas of fibronectin-containing tissue (Figure 6c, d), which must have been mouse in origin, because no areas of the tumour were stained with the monoclonal antibody IST2 (data not shown). The islands of tumour cells were surrounded by an almost continuous basement membrane-like pattern of laminin staining (Figure 6e, f), but the tumour itself was not

**Figure 4** Analysis of human keratin polypeptides. (a) Coomassie blue-stained SDS-polyacrylamide gel of keratin fractions. (b) Immunoblot stained with AE1 monoclonal anti-keratin antibody. (c) Immunoblot stained with AE3 monoclonal. Lane 1, adult epidermis; lane 2, cultured adult keratinocytes; lane 3, cultured foetal keratinocytes; lane 4, SV6-1 Bam/HFK cells at passage 12; lane 5, SV6-1 Bam/HFK at passage 48; lane 6, tumour produced by SV6-1 Bam/HFK at passage 48; lane 7, cells recultured from SV6-1 Bam/HFK tumour. On the left the keratin polypeptides are identified by their apparent molecular weights ($Mr \times 10^{-3}$). Figures in square brackets indicate the probable identities of the keratins using the numerical nomenclature of Moll *et al.* (1982); see Sun and Green (1978). Dots in (b), lane 6, identify novel 55 Kd and 45 Kd AE1-positive keratins.

encapsulated by a basement membrane in any of the tumour specimens examined (data not shown).

### Discussion

The SV40 transformed human epidermal keratinocyte cell line described in this paper undergoes spontaneous progression to a malignant phenotype, which makes this line unique, we believe, but in many other respects it resembles previously described SV40 transformed keratinocyte lines (Steinberg & Defendi, 1979, 1985; Taylor-Papadimitriou *et al.*, 1982; Banks-Schlegel & Howley, 1983; Rhim *et al.*, 1985; Hronis *et al.*, 1984; Bernard *et al.*, 1985; Banks-Schlegel & Rhim, 1986; Edelman *et al.*, 1985; Defendi *et al.*, 1982): (1) The line went through no apparent 'crisis' period during its establishment; (2) The line demonstrated decreased growth requirements when compared with normal keratinocytes; (3) The cells were less differentiated than normal keratinocytes, as shown by their lack of stratification *in vitro*, their reduced ability to undergo terminal differentiation when placed in suspension (Parkinson *et al.*, 1983, 1984), and their altered response to tumour promoters (Parkinson *et al.*, 1983, 1984); (4) our SV40 transformed keratinocytes retained the ability to produce extracellular matrix components, and at early passage levels produced increased amounts of fibronectin (Brown & Parkinson, 1984).

The pattern of keratin expression by the SV6-1 Bam/HFK cells had features in common with previous reports. Firstly, the transformants retained the expression of the keratins expressed by normal keratinocytes *in vitro* i.e. 58 Kd, 56 Kd, 50 Kd, 48 Kd and 46 Kd as found for some other SV40 transformants (Banks-Schlegel & Howley, 1983; Bernard *et al.*, 1985; Banks-Schlegel & Rhim, 1986), although expression of the 48 Kd and 50 Kd species was markedly reduced (Figure 4). Secondly, as has been found by other investigators (Taylor-Papadimitriou *et al.*, 1982; Banks-Schlegel & Howley, 1983; Hronis *et al.*, 1984; Steinberg & Defendi, 1985; Bernard *et al.*, 1985; Banks-Schlegel & Rhim, 1986), the expression of keratins normally found in simple epithelia (54 Kd and 52 Kd; Figure 4) was increased in our SV40 transformed keratinocytes, although we could find no significant difference in 40 Kd keratin expression between normal and transformed cells (Figure 4). In the tumour, the pattern of keratin expression closely resembled that of cultured keratinocytes (Figure 4) i.e. the tumour expressed a 'hyperproliferative' keratin pattern, as has been found in human SCCs (Weiss *et al.*, 1984). On re-establishing the tumour cells *in vitro*, the keratin pattern reverted to one similar to that of cultured SV6-1 Bam/HFK cells, once again emphasising how keratin expression is modulated by the cell's external environment (Eichner *et al.*, 1984; Fuchs & Green, 1981; Doran *et al.*, 1980). Although some differentiation was evident in the tumour, demonstrated by the production of keratin 'pearls' (Figure 3b), no 67 Kd keratin (characteristic of fully keratinized epidermis (Eichner *et al.*, 1984)) was detected. This could have merely reflected the very low level of differentiation as compared to normal epidermis, or alternatively the area of tumour used for keratin analysis may have been particularly undifferentiated. Two novel AE1 positive keratin bands (45 Kd and 55 Kd) were detected in the tumour, but not in any of the cultured cells (Figure 4).

In this paper we have extended our previous *in vitro* (Brown & Parkinson, 1984) studies of the keratinocyte extracellular matrix to the *in vivo* situation. Interestingly, low passage SV6-1 Bam/HFK keratinocytes expressed fibronectin in the cysts (Figure 5c), but no human fibronectin could be detected in the SCC tumours formed by the high passage cells (data not shown), although the tumours contained large amounts of mouse fibronectin (Figure 6d). Thus the reduction in fibronectin production which occurs as SV6-1 Bam/HFK keratinocytes are passaged *in vitro* (Brown & Parkinson, 1984) is maintained *in vivo*. In contrast, both high and low passage cells synthesized laminin *in vitro*, and although we could not definitively show production of laminin by SV6-1 Bam/HFK keratinocytes *in vivo* (as no human specific antibody was available) the close proximity of T-antigen positive cells and areas of laminin staining in both the cysts (Figure 5d, e) and tumours (Figure 6e, f) suggests that both the low- and high-passage cells may produce laminin *in vivo*. Thus it appears that at least some basement membrane components are produced in SV6-1 Bam/HFK induced nude mouse tumours, which is similar to the results reported by Gusterson *et al.* (1984) for human SCCs.

The truly novel result of our work has been the finding that after extensive passaging *in vitro* (corresponding to approximately 200 population doublings) SV6-1 Bam/HFK

**Figure 5** Indirect immunofluorescence of a nude mouse cyst formed by SV6-1 Bam/HFK at passage 17 (8 days post-inoculation). (a) SV40 T-antigen; (b) Keratin; (c) Human fibronectin (IST2 monoclonal antibody). (d) and (e), double staining of the same field; (d), SV40 T-antigen; and (e) Laminin. Bars = 100 μm for (a–c). × 120 and 20 μm for (d), (e). × 500.

keratinocytes formed malignant tumours when inoculated into athymic (nude) mice. Similarly, it has been reported that many SV40 transformed rodent cells require extensive passaging before they become tumourigenic (Tevethia, 1980). Several *in vitro* phenotypic changes were associated with the development of malignant potential in SV6-1 Bam/HFK: (1) An alteration in cellular morphology (Figure 1); (2) Development of a cytogenetically marked stem cell line (Table I); and (3) A reduction in fibronectin production (Brown & Parkinson, 1984). These alterations could be explained by the development of an altered subpopulation of transformants with a selective growth advantage, which eventually outgrew the non-malignant transformants.

Whether any of these changes in *in vitro* phenotype were a cause or a consequence of the development of malignancy, they clearly indicate that a second event (or more) had occurred after the initial establishment of the cell line. This second event could possibly have been a re-arrangement of integrated viral sequences, although the lack of an origin of replication in the SV6-1 DNA used (Gluzman *et al*., 1980) means that the integrated viral sequences would be unable to undergo any T-antigen directed replication that could promote excision and re-integration of viral DNA. Indeed Southern blotting data (Byrd & Gallimore, unpublished data) have shown no major differences in the pattern of integrated SV40 sequences between low- and high-passage SV6-1 Bam/HFK cells (and additionally this confirms that the squamous cell carcinomas were produced by SV6-1 Bam/HFK cells).

Other likely candidates for possible second events are the activation of cellular proto-oncogenes and/or loss of gene products capable of malignancy suppression. In particular *ras* oncogenes have been implicated in the development of epidermal malignancies (reviewed in Balmain, 1985) and infection of mouse keratinocytes with *ras*-containing retroviruses has been shown to induce alterations in their differentiation programme and growth factor requirements (Yuspa *et al*., 1985; Weissman & Aaronson, 1985). In addition Rhim *et al*. (1985) have recently demonstrated that human keratinocytes immortalized with an adenovirus 12-SV40 hybrid virus could then be converted to a malignant phenotype by addition of Kirsten murine sarcoma virus, a *ras*-containing virus. However, Rhim *et al*. (1986) have also found that Ad 12-SV40 immortalised human keratinocytes can become malignant after treatment with chemical carcinogens *in vitro*, but in this case they found no evidence

**Figure 6** Indirect immunofluorescence of a nude mouse tumour formed by SV6-1 Bam/HFK at passage 47 (60 days post-inoculation). (a), (c) and (e), SV40 T-antigen; (b) keratin; (d), fibronectin; (f), laminin. (a-b), (c-d) and (e-f) are pairs showing the same fields double stained for T-antigen and keratin or fibronectin or laminin. Bars = 20 μm. × 500.

to suggest *ras* activation. We are now investigating whether there is any evidence of *ras* gene activation in high passage SV6-1 Bam/HFK cells. Spontaneous activation of the N-*ras* proto-oncogene has been previously demonstrated following *in vitro* cultivation of a human teratocarcinoma cell line (Tainsky *et al.*, 1984).

Previous reports have indicated that other SV40 transformed human keratinocytes are non-tumourigenic (Chang, 1986; Banks-Schlegel & Howley, 1983; Hronis *et al.*, 1984), even though some have been shown to form short-lived cysts which resembled human SCCs (Banks-Schlegel & Howley, 1983). The development of malignancy in the SV6-1 Bam/HFK line required extensive *in vitro* passaging, and one possibility is that other SV40 transformants have simply not been passaged sufficiently to accumulate secondary events necessary to produce malignancy. The difference in our line is probably not due to the use of foetal keratinocytes since, from a number of independently derived SV40 transformed human keratinocyte cell lines from post-neonatal donors with an inherited susceptibility to cancer, two eventually acquired a malignant phenotype on passage (Gallimore, Stacey & Taylor, unpublished data).

The evolution of a cytogenetically marked clonal population was clearly a major feature of the progression of SV6-1 Bam/HFK cells. Of the five marker chromosomes identified in the high passage clone only markers 1 and 2 were retained in the nude mouse derived SCC lines. Although we have been unable to identify unequivocally the origins of these markers their retention in the tumours may indicate that genes mapping on these abnormal markers made a contribution to the genesis of a malignant phenotype. In addition, the cytogenetic changes to autosomes 3 and 11 may also be of significance. A number of human tumours have been shown to have deletions or re-arrangements of the *p* arms of chromosome 3 similar to SV6-1 Bam/HFK (Figure 2), including tumours of epithelial origin (Yoshida *et al.*, 1985). Interestingly, Teysseir *et al.* (1986) recently showed that in two renal cell carcinomas the deletion of 3*p* resulted in the interstial translocation of the proto-oncogene c-*raf* 1 from 3*p* 25 to 3*p* 14. At least two proto-oncogenes, c-Ha-*ras* 1 (11*p* 15, de Martinville & Franke, 1983) and c-*ets* (11*q* 23–24 De Taisne *et al*., 1984) have been mapped to chromosome 11, as has the locus associated with Wilm's tumour (11*p* 13, Koufos *et al*., 1984). Genetic change to the single copy of chromosome 11 in the high passage SV6-1 Bam/HFK clone at any of these genetic loci may have generated the malignant phenotype. Saxon *et al*. (1986) have recently shown that chromosome 11 can suppress the malignant phenotype of HeLa cells. Clearly the possible involvement of the loss of one copy of autosome 11 in the malignant phenotype of SV6-1 Bam/HFK cell could be resolved by the development of somatic cell hybrids between the fibroblast hybrid ESH15(T1) and SV6-1 Bam/HFK SCC, in an analogous manner to Saxon *et al*.

The results described in this paper, together with a previous report (Tainsky *et al*., 1984) indicate that profound genetic changes may occur spontaneously during the *in vitro* cultivation of human cells, and therefore caution is indicated in interpreting the results from so-called '2-stage' *in vitro* models where multiple genes or agents have been used, since conversion of SV6-1 transformed normal keratinocytes to a malignant phenotype may have represented several spontaneous events.

We are most grateful to Mr R. Barthakur, Mr P. Biggs, Mr P. Grabham, Mr P. Reeve, Miss V. Nash and Mrs E. Fletcher for skilled technical assistance, Mrs S. Williams for the photography, Miss D. Williams for typing the manuscript, Drs P.J. Byrd, C. Paraskeva and A.M.R. Taylor for their helpful comments on the manuscript. This work was supported by the Cancer Research Campaign, of which P.H.G. is a Life Fellow.

### References

BALMAIN, A. (1985). Transforming *ras* oncogenes and multistage carcinogenesis. *Br. J. Cancer*, **51**, 1.

BANKS-SCHLEGEL, S.P. & HOWLEY, P.M. (1983). Differentiation of human epidermal cells transformed by SV40. *J. Cell. Biol*., **96**, 330.

BANKS-SCHLEGEL, S.P. & RHIM, J.S. (1986). Keratin expression of both chemically and virally transformed human epidermal keratinocytes during the process of neoplastic conversion. *Carcinogenesis*, **7**, 153.

BERNARD, B.A., ROBINSON, S.M., SEMAT, A. & DARMON, M. (1985). Reexpression of fetal characters in Simian Virus 40-transformed human keratinocytes. *Cancer Res*., **45**, 1707.

BROWN, K.W. & PARKINSON, E.K. (1984). Extracellular matrix components produced by SV40-transformed human epidermal keratinocytes. *Int. J. Cancer*, **33**, 257.

BROWN, K.W. & PARKINSON, E.K. (1985). Alteration of the extracellular matrix of cultured human keratinocytes by transformation and during differentiation. *Int. J. Cancer*, **35**, 799.

BURNETT, T.S. & GALLIMORE, P.H. (1983). Establishment of a human keratinocyte cell line carrying complete human papillomavirus type 1 genomes: lack of vegetative viral DNA synthesis upon keratinization. *J. Gen. Virol*., **64**, 1509.

CHANG, S.E. (1986). *In vitro* transformation of human epithelial cells. *Biochim. Biophys. Acta*, **823**, 161.

DEFENDI, V., NAIMSKI, P. & STEINBERG, M.L. (1982). Human cells transformed by SV40 revisited: The epithelial cell. *J. Cell. Physiol*., Suppl. **2**, 131.

DE MARTINVILLE, B. & FRANKE, U. (1983), The c-Ha-*ras* 1, insulin and β-globin loci map outside the deletion associated with aniridia-Wilms' tumour. *Nature*, **305**, 641.

DE TAISNE, C., GEGONNE, A., STEHELIN, D., BERNHEIM, A. & BERGER, R. (1984). Chromosomal localization of the human proto-oncogene c-*ets*. *Nature*, **310**, 581.

DORAN, T.I., VIDRICH, A. & SUN, T.-T. (1980). Intrinsic and extrinsic regulation of the differentiation of skin, corneal and esophageal epithelial cells. *Cell*, **22**, 17.

EDELMAN, B., STEINBERG, M.L. & DEFENDI, V. (1985). Changes in fibronectin synthesis and binding distribution in SV40-transformed human keratinocytes. *Int. J. Cancer*, **35**, 219.

EICHNER, R., BONITZ, P. & SUN, T.-T. (1984). Classification of epidermal keratins according to their immunoreactivity, isoelectric point, and mode of expression. *J. Cell. Biol*., **98**, 1388.

FUCHS, E. & GREEN, H. (1981). Regulation of terminal differentiation of cultured human keratinocytes by vitamin A. *Cell*, **25**, 617.

GALLIMORE, P.H. & RICHARDSON, C.R. (1973). An improved banding technique exemplified in the karyotypic analysis of two strains of rat. *Chromosoma*, **41**, 259.

GEIGER, P.J. & BESSMAN, S.P. (1972). Protein determination by Lowry's method in the presence of sulphydryl reagents. *Anal. Biochem*., **49**, 467.

GLUZMAN, Y., FRISQUE, R.J. & SAMBROOK, J. (1980). Origin-defective mutants of SV40. Cold Spring Harb. *Symp. Quant. Biol*., **44**, 293.

GRAHAM, F.L. & VAN DER EB, A.J. (1973). A new technique for the assay of infectivity of human adenovirus 5 DNA. *Virology*, **52**, 456.

GUSTERSON, B.A., WARBURTON, M.J., MITCHELL, D., KRAFT, N. & HANCOCK, W.W. (1984). Invading squamous cell carcinoma can retain a basal lamina. An immunohistochemical study using a monoclonal antibody to type IV collagen. *Lab. Invest*., **51**, 82.

HRONIS, T.S., STEINBERG, M.L., DEFENDI, V. & SUN, T.-T. (1984). Simple epithelial nature of some Simian-virus 40-transformed human epidermal keratinocytes. *Cancer Res*., **44**, 5797.

JACKSON, P. & THOMPSON, R.J. (1984). The immunodetection of brain proteins blotted onto nitrocellulose from fixed and stained two-dimensional polyacrylamide gels. *Electrophoresis*, **5**, 35.

JOHNSON, G.D., DAVIDSON, R.S., MCNAMEE, K.C., RUSSELL, G., GOODWIN, D. & HOLBOROW, E.J. (1982). Fading of immunofluorescence during microscopy: a study of the phenomena and its remedy. *J. Immunol. Meth*., **55**, 231.

KOUFOS, A., HANSEN, M.F., LAMPKIN, B.C. & 4 others (1984). Loss of alleles at loci on human chromosome 11 during genesis of Wilms tumour. *Nature*, **309**, 170.

LAEMMLI, U.K. (1970). Cleavage of structural proteins during the assembly of the head of bacteriophage T4. *Nature*, **227**, 680.

LAND, H., PARADA, L.F. & WEINBERG, R.A. (1983). Tumourigenic conversion of primary embryo fibroblasts requires at least two co-operating oncogenes. *Nature*, **304**, 596.

MOLL, R., FRANKE, W.W., SHILLER, D.L., GEIGER, B. & KREPLER, R. (1982). The catalog of human cytokeratins: Patterns of expression in normal epithelia, tumours and cultured cells. *Cell*, **31**, 11.

PARKINSON, E.K., GRABHAM, P. & EMMERSON, A. (1983). A subpopulation of cultured human keratinocytes which is resistant to the induction of terminal differentiation-related changes by phorbol. 12-myristate, 13-acetate: evidence for an increase in the resistant population following transformation. *Carcinogenesis*, **4**, 857.

PARKINSON, E.K., PERA, M.R., EMMERSON, A. & GORMAN, P.A. (1984). Differential effects of complete and second-stage tumour promoters in normal but not transformed human and mouse keratinocytes. *Carcinogenesis*, **5**, 1071.

RHEINWALD, J.G. & BECKETT, M.A. (1980). Defective terminal differentiation in culture as a consistent and selectable character of malignant human keratinocytes. *Cell*, **22**, 629.

RHEINWALD, J.G. & BECKETT, M.A. (1981). Tumorigenic keratinocyte lines requiring anchorage and fibroblast support cultured from human squamous cell carcinoma. *Cancer Res*., **41**, 1657.

RHEINWALD, J.G. & GREEN, H. (1975). Serial cultivation of strains of human epidermal keratinocytes: the formation of keratinizing colonies from single cells. *Cell*, **6**, 331.

RHEINWALD, J.G. & GREEN, H. (1977). Epidermal growth factor and the multiplication of cultured human epidermal keratinocytes. *Nature*, **265**, 421.

RHIM, J.S., FUJITA, J., ARNSTEIN, P. & AARONSON, S.A. (1986). Neoplastic conversion of human keratinocytes by adenovirus 12-SV40 virus and carcinogens. *Science*, **232**, 385.

RHIM, J.S., JAY, G., ARNSTEIN, P., PRICE, F.M., SANFORD, K.K. & AARONSON, S.A. (1985). Neoplastic transformation of human epidermal keratinocytes by Ad 12-SV40 and Kirsten Sarcoma viruses. *Science*, **227**, 1250.

RULEY, H.E. (1983). Adenovirus early region 1A enables viral and cellular transforming genes to transform primary cells in culture. *Nature*, **304**, 602.

RUPNIAK, H.T., ROWLATT, C., LANE, E.B. & 5 others (1985). Characteristics of four new human cell lines derived from squamous cell carcinomas of the head and neck. *J. Natl Cancer Inst.*, **75**, 621.

SAXON, P.J., SRIVATSAN, E.S. & STANBRIDGE, E.J. (1986). Introduction of human chromosome 11 via microcell transfer controls tumourigenic expression of HeLa cells. *EMBO J.*, **5**, 3461.

STEINBERG, M.L. & DEFENDI, V. (1979). Altered pattern of growth and differentiation in human keratinocytes infected by Simian Virus 40. *Proc. Natl. Acad. Sci. USA*, **76**, 801.

STEINBERG, M.L. & DEFENDI, V. (1985). Altered pattern of keratin synthesis in human epidermal keratinocytes transformed by SV40. *J. Cell. Physiol.*, **123**, 117.

SUN, T.-T. & GREEN, H. (1978). Keratin filaments of cultured human epidermal cells. *J. Biol. Chem.*, **253**, 2053.

TAINSKY, M.A., COOPER, C.S. & GIOVANELLA, B.C. (1984). An activated ras N gene: detected in late but not early passage human PA1 teratocarcinoma cells. *Science*, **225**, 643.

TAYLOR-PAPADIMITRIOU, J., PURKIS, P., LANE, E.B., McKAY, I.A. & CHANG, S.E. (1982). Effects of SV40 transformation on the cytoskeleton and behavioural properties of human keratinocytes. *Cell Differentiation*, **11**, 169.

TEVETHIA, S.S. (1980). Immunology of Simian virus 40. In *Viral Oncology*, Klein, G. (ed) p. 581. Raven Press: New York.

TEYSSEIR, J.R., HENRY, I., DOZIER, C., FERRE, D., ADNET, J.J. & PLUOT, M. (1986). Recurrent deletion of the short arm of chromosome 3 in human renal cell carcinoma: Shift of the c-*raf*-1 locus. *J. Natl Cancer Inst.*, **77**, 1187.

WEISS, R.A., EICHNER, R. & SUN, T.-T. (1984). Monoclonal antibody analysis of keratin expression in epidermal diseases: A 48- and 56-K dalton keratin as molecular markers for hyperproliferative keratinocytes. *J. Cell. Biol.*, **98**, 1397.

WEISSMAN, B. & AARONSON, S.A. (1985). Members of the src and ras oncogene families supplant the epidermal growth factor requirement of BALB/MK-2 keratinocytes and induce distinct alterations in their terminal differentiation program. *Mol. Cell. Biol.*, **5**, 3386.

WOODCOCK-MITCHELL, J., EICHNER, R., NELSON, W.G. & SUN, T.-T. (1982). Immunolocalization of keratin polypeptides in human epidermis using monoclonal antibodies. *J. Cell. Biol.*, **95**, 580.

WU, Y.-J. & RHEINWALD, J.G. (1981). A new small (40 Kd) keratin filament protein made by some cultured human squamous cell carcinomas. *Cell*, **25**, 657.

YOSHIDA, M.A., OCHI-TAKEUCHI, H., GIBAS, Z. & SANDBERG, A.A. (1985). Updating of chromosome changes in renal cell carcinoma. *Proc. Am. Assoc. Cancer Res.*, **26**, 31 (*abstract*).

YUSPA, S.H., KILKENNY, A.E., STANLEY, J. & LICHTI, U. (1985). Keratinocytes blocked in phorbol ester-responsive early stage of terminal differentiation by sarcoma viruses. *Nature*, **314**, 459.

ZARDI, L., CARNEMOLLA, B., SIRI, A., SANTI, L. & ACCOLLA, R.S. (1980). Somatic cell hybrids producing antibodies specific to human fibronectin. *Int. J. Cancer*, **25**, 325.