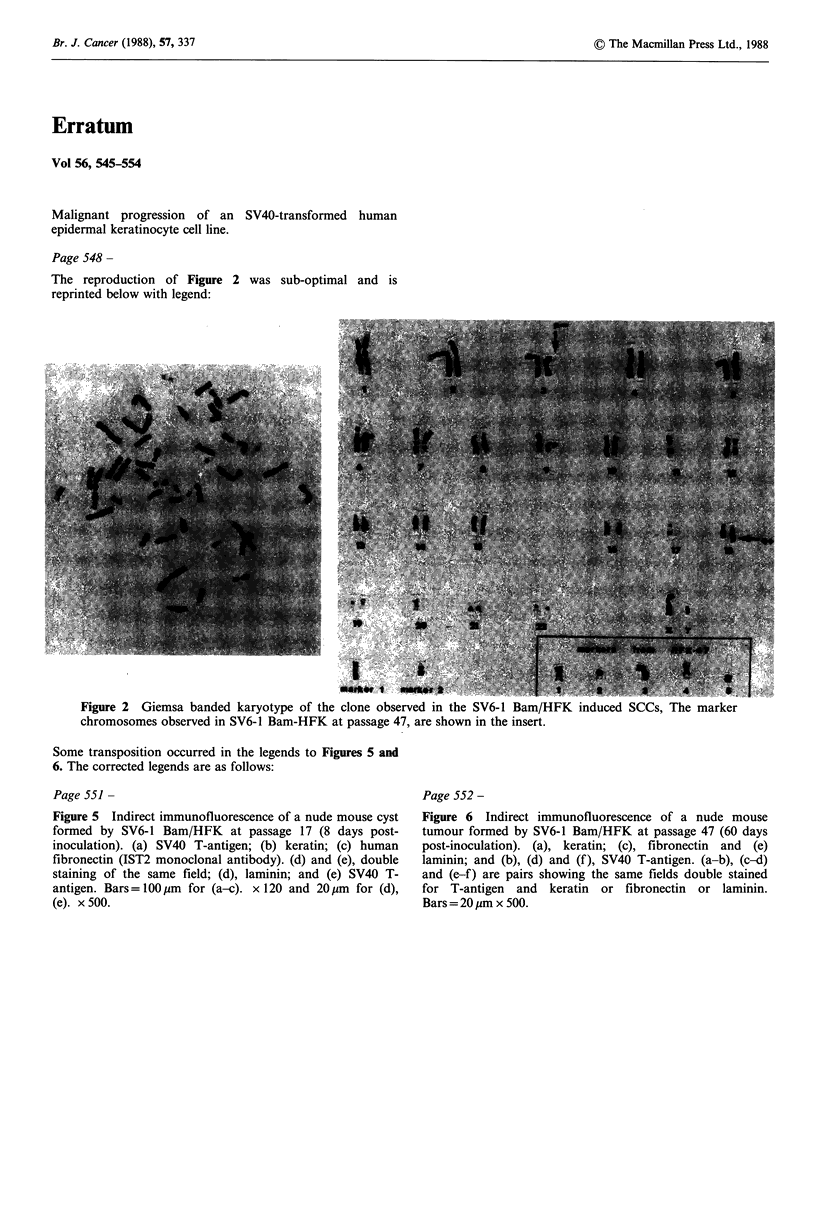# Erratum

**Published:** 1988-03

**Authors:** 

## Abstract

**Images:**


© The Macmillan Press Ltd., 1988

### Erratum

**Vol 56, 545–554**

Malignant progression of an SV40-transformed human epidermal keratinocyte cell line.

*Page 548 –*

The reproduction of **Figure 2** was sub-optimal and is reprinted below with legend:

**Figure 2** Giemsa banded karyotype of the clone observed in the SV6-1 Bam/HFK induced SCCs, The marker chromosomes observed in SV6-1 Bam-HFK at passage 47, are shown in the insert.

Some transposition occurred in the legends to **Figures 5 and 6**. The corrected legends are as follows:

*Page 551 –*

**Figure 5** Indirect immunofluorescence of a nude mouse cyst formed by SV6-1 Bam/HFK at passage 17 (8 days post-inoculation). (a) SV40 T-antigen; (b) keratin; (c) human fibronectin (IST2 monoclonal antibody). (d) and (e), double staining of the same field; (d), laminin; and (e) SV40 T-antigen. Bars = 100 $\mu$m for (a–c). $\times 120$ and 20 $\mu$m for (d), (e). $\times 500$.

*Page 552 –*

**Figure 6** Indirect immunofluorescence of a nude mouse tumour formed by SV6-1 Bam/HFK at passage 47 (60 days post-inoculation). (a), keratin; (c), fibronectin and (e) laminin; and (b), (d) and (f), SV40 T-antigen. (a–b), (c–d) and (e–f) are pairs showing the same fields double stained for T-antigen and keratin or fibronectin or laminin. Bars = 20 $\mu$m $\times$ 500.